# Supplementary material for: Gold Nanoparticles as a Platform for Delivery of Immunogenic Peptides to THP-1 Derived Macrophages: Insights into Nanotoxicity
Source: Vaccines (Basel). 2025 Jan 24;13(2):119. doi: 10.3390/vaccines13020119 (PMC11860437; doi:10.3390/vaccines13020119)
Supplement: Supplementary file 1 [file vaccines-13-00119-s001.zip › vaccines-3399275-supplementary.pdf]

Supplementary Material

Gold Nanoparticles as a Platform for Delivery of Immunogenic Peptides to THP-1 Derived Macrophages: Insights into Nanotoxicity

Eduardo Zúñiga [1], Braulio Contreras-Trigo [1], Jorge Buchert [2], Fabián Sáez-Ahumada [2], Leonardo Hernández [2], Víctor Fica [1], Estefania Nova-Lamperti [2], Bostjan Kobe [3], Fanny Guzmán [4], Víctor Diaz-García [1], Enrique Guzmán-Gutiérrez [2] and Patricio Oyarzún [1,*]

[1] Facultad de Ingeniería, Arquitectura y Diseño, Universidad San Sebastián, Concepción 4081339, Chile; ezunigaz@docente.uss.cl (E.Z.); bcontrerast@docente.uss.cl (B.C.-T.); vfical@correo.uss.cl (V.F.); victor.diazg@uss.cl (V.D.-G.); patricio.oyarzun@uss.cl (P.O.)
[2] Departamento de Bioquímica Clínica e Inmunología, Facultad de Farmacia, Universidad de Concepción, Concepción 4070386, Chile; jbuchert2016@udec.cl (J.B.); fabsaez@udec.cl (F.S.-A.); lehernandez2019@udec.cl (L.H.); enova@udec.cl (E.N.-L.); eguzman@udec.cl (E.G.-G.)
[3] School of Chemistry and Molecular Biosciences, Institute for Molecular Bioscience, and Australian Infectious Diseases Research Centre, The University of Queensland, Brisbane, QLD 4072, Australia; b.kobe@uq.edu.au
[4] Núcleo Biotecnología Curauma, Pontificia Universidad Católica de Valparaíso, Valparaíso 2373223, Chile; fanny.guzman@pucv.cl
* Correspondence: patricio.oyarzun@uss.cl

Supplementary methods 1. Procedure to determine the peptide surface coverage (densities).

AuNPs concentration was determined according to the microsynthesis method previously described by our group [1]. The procedure is briefly described in equation 1, where D corresponds to the diameter of AuNPs and ε is the molar extinction coefficient; these values are subsequently employed using the Beer-Lambert law (equation 2) to determine AuNPs concentration, where A is the maximum absorbance at the surface plasmon resonance (SPR band), c is the AuNPs concentration and b is path length of light (in cm).

The next example for Au23 considers a path length of 0.3 cm (corresponding to the dispense of 100 μL of the sample in 96 well plates) and an absorbance value at the SPR (520 nm) of 1.216.

$$Ln_{\varepsilon} = 3.321111\ Ln\ D\ +\ 10.80505 \tag{1}$$

$$\varepsilon\ =\ e^{(3.321111\ Ln\ 23+10.80505)}$$

$$\varepsilon\ =\ 1.64x10^{9}$$

$$A\ =\ \varepsilon\ c\ b \tag{2}$$

$$c\ =\ \frac{A}{\varepsilon\ b}$$

$$c\ =\ \frac{1.216}{1.64x10^{9}x0.3}$$

$$c\ =\ 2.47\cdot 10^{-9}\ M = 2.47\ nM$$

The same procedure was employed to determine the Au68 concentration of 0.054 nM (absorbance at 539 nm = 0.972)

The area of AuNPs was calculated according to equation 3, where A is area of the AuNPs, and r is the radius of the AuNPs (determined by TEM). Then, the peptide nmoles for each molar ratio (per AuNPs size) were determine by equation 4. Finally, the surface in nmol-peptide/nm^2 was determined by equation 5.

Thus, the next calculation corresponds to the determination of Au23 (nmoles) at a molar ratio of AuNPs:peptide 1:100,000, considering the functionalization of 2.0 mL of 8.23 nM Au23.

$$A_{s-AuNPs} = 4\pi r^{2} \tag{3}$$

$$A_{s-AuNPs}\ = 4\pi 11.5^{2}$$

$$A_{s-AuNPs} =\ 1661.9nm^{2}$$

$$nmol\ peptide\ =\ nmol\ AuNPs\ x\ proportion \tag{4}$$

$$nmol\ peptide\ =\ \frac{2.47\ nmol\ x\ 2.0\ mL}{1{,}000\ mL}\ x\ 100{,}000$$

$$nmol\ peptide\ =\ 494\ nmol$$

$$nmol-peptide/nm^2 = \frac{nmol\ peptide}{A_{S-AuNPs}} \quad (5)$$

$$nmol-peptide/nm^2 = \frac{494\ nmol}{1{,}661.9\ nm^2}$$

$$nmol-peptide/nm^2 = 0.30\ nmol/nm^2$$

The table below summarizes the calculations to determine the peptide surface coverage (densities) for AuNPs of both sizes (Au23 and Au68):

**Parameter**	**Au23**	**Au68**
AuNP size by TEM (nm)	23	68
AuNP surface (nm^2)	1661.9	14526.8
AuNPs concentration (nM)	2.47	0.054
AuNPs in 2.0 mL (nmol)	0.00494	0.000108
peptide coverage 0.75 (nmol)	370.5	3238.5
peptide coverage 1.0 (nmol)	494.0	4318.1
peptide coverage 1.25 (nmol)	617.5	5397.6
Surface density - coverage 0.75 (nmol-peptide/nm^2)	0.22	0.22
Surface density - coverage 1.0 (nmol-peptide/nm^2)	0.30	0.30
Surface density - coverage 1.25 (nmol-peptide/nm^2)	0.37	0.37

References

[1] Díaz-García, V.; Haensgen, A.; Inostroza, L.; Contreras-Trigo, B.; Oyarzun, P. Novel Microsynthesis of High-Yield Gold Nanoparticles to Accelerate Research in Biosensing and Other Bioapplications. Biosensors (Basel) 2023, 13, 992, doi:10.3390/bios13120992.

Table S1. Predivac-3.0 predictions. Epitope discovery mode was employed to predict CD4+ T-cell epitopes and population coverage potentially delivered by peptide 1 (CALNNKKPKYVKQNTLKLAT) for the Chilean population.

nonamer ID	Predicted CD4+ T-cell epitope	Predicted coverage (%)	Alleles
**1**	YVKQNTLKL	93.1461	HLA-DRB1*01:01, HLA-DRB1*01:02, HLA-DRB1*04:01, HLA-DRB1*04:02, HLA-DRB1*04:03, HLA-DRB1*04:04, HLA-DRB1*04:05, HLA-DRB1*04:07, HLA-DRB1*07:01, HLA-DRB1*08:01, HLA-DRB1*08:02, HLA-DRB1*09:01, HLA-DRB1*10:01, HLA-DRB1*11:01, HLA-DRB1*12:01, HLA-DRB1*13:01, HLA-DRB1*13:02, HLA-DRB1*13:03, HLA-DRB1*14:02, HLA-DRB1*14:06, HLA-DRB1*15:01, HLA-DRB1*15:02, HLA-DRB1*16:01, HLA-DRB1*16:02
**2**	VKQNTLKLA	22.8940	HLA-DRB1*03:01, HLA-DRB1*11:02, HLA-DRB1*11:03, HLA-DRB1*11:04, HLA-DRB1*14:01

Table S2. Instrumentally derived parameters characterizing Au23 and Au68 nanoconjugates.

Analysis	Item	Au23		Au68	
		Free AuNPs	p2-Au23 (coverage 1.25)	Free AuNPs	p2-Au68 (coverage 1.25)
TEM	Average (nm)	23.4	25.4	68.2	64.0
	SD	2.5	1.9	8.3	16.3
	CV (%)	10.7	7.5	12.2	25.5
	Range (nm)	18 - 30	21 - 38	48 - 86	22 - 93
	Number of spheres	425	390	25	45
	Number of total NPs	551	610	50	88
	Spheres/NPs (%)	76	64	50	51
	p-value	0.0001		0.2395	
	Significant differences ($p < 0.05$)	Yes		No	
Spectrogram	SPR (nm)	520 - 521	523 - 524	539	541 - 545
	O. D. (dilution)	0.608 (1/2)	0.381 (without dil)	0.486 (1/2)	0.366 (without dil)
	Concentration (nM)	2.470	2.118	0.054	0.034
pZ	Voltage (mV)	-42.64	30.62	-40.45	38.77
	SD	0.71	0.37	1.83	0.75
	RSD (%)	1.67	1.21	4.52	1.93

Table S3. Chemical properties of the four peptides conjugated to gold nanoparticles.

Item	Peptide 1	Peptide 2	Peptide 3	Peptide 4
Number of residues	20	28	23	31
Molecular weight (g/mol)	2275,74	3441,16	2384,78	3550,2
Isoelectric point	10,53	12,36	10,09	12,34
Net charge at pH 7.0	4,95	11,95	2,95	9,95
Net charge at pH < 7.0	5 a 6	> 12	3 a 4	> 10
Average hydrophilicity	0,19	0,89	-0,10	0,61
hydrophilic residues / total number residues (%)	45	61	30	48
Number residues with positive charge	6	13	4	11

Peptide 1: CALNNKKPKYVKQNTLKLAT
Peptide 2: CALNNKKPKYVKQNTLKLATRKKRQRRR
Peptide 3: CALNNGPGPGPKYVKQNTLKLAT
Peptide 4: CALNNGPGPGPKYVKQNTLKLATRKKRQRRR

Table S4. Comparison between the size and the number of gold atoms present in AuNPs of 23 nm and 68 nm.

AuNP	Diameter (nm)	AuNPs surface (nm2)	Gold atoms Number per AuNPs
Au23	23	1661,9	375912
Au68	68	14526,8	9714697
Ratio Au68/Au23	2,96	8,74	25,84

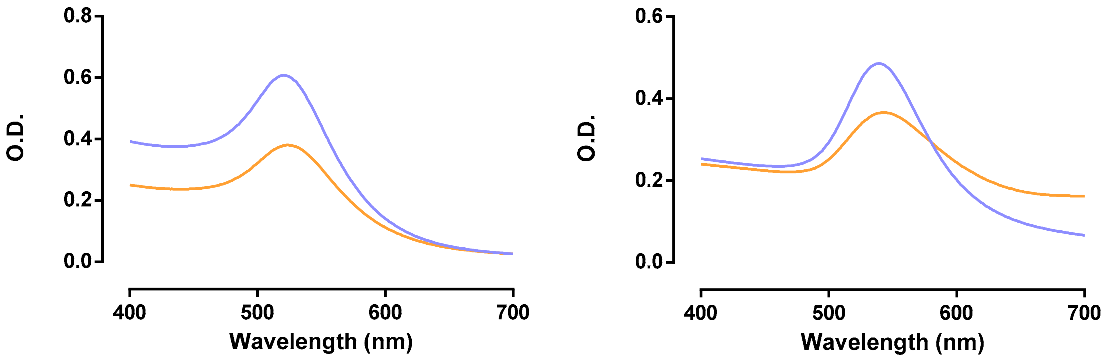

Figure S1. Visible spectrum of (A) Au23 and (B) Au68. The spectra show the surface plasmon resonance (SPR) band for free AuNPs (blue line) and p2-AuNPs nanoconjugates (red line).

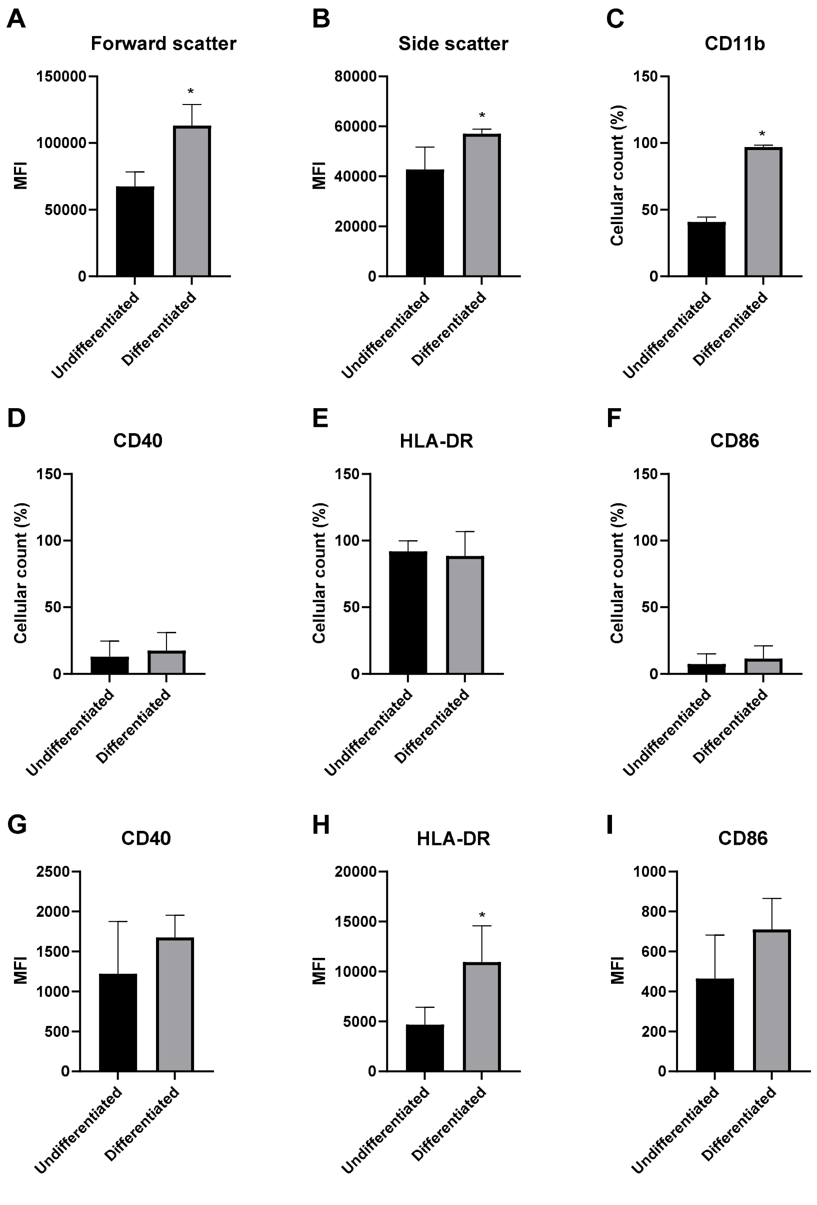

Figure S2. Differentiation markers of THP-1 cells. THP-1 monocytes (undifferentiated, black bar) were differentiated to THP-1 macrophages (differentiated, gray bar) using PMA and LPS. It evaluated (A) Forward Scatter, (B) Side Scatter; Cellular count for (C) CD11b, (D) CD40, (E) HLA-DR, and (F) CD86; and MFI for (G) CD40, (H) HLA-DR, and (I) CD86. *P<0,05 in comparison with undifferentiated cells (n = 3).

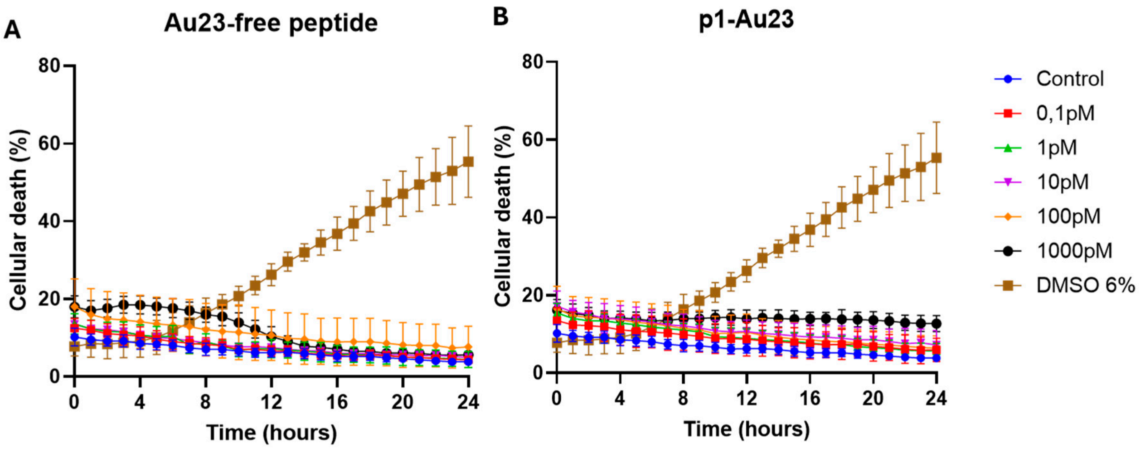

Figure S3. Effect of AuNPs concentration (0.1 to 1000 pM) on percentage of macrophages THP-1 death. (A) Au23-free peptide and (B) p1-Au23. Control cells were not exposed to AuNPs. As a positive control of cellular death was 6% of DMSO.

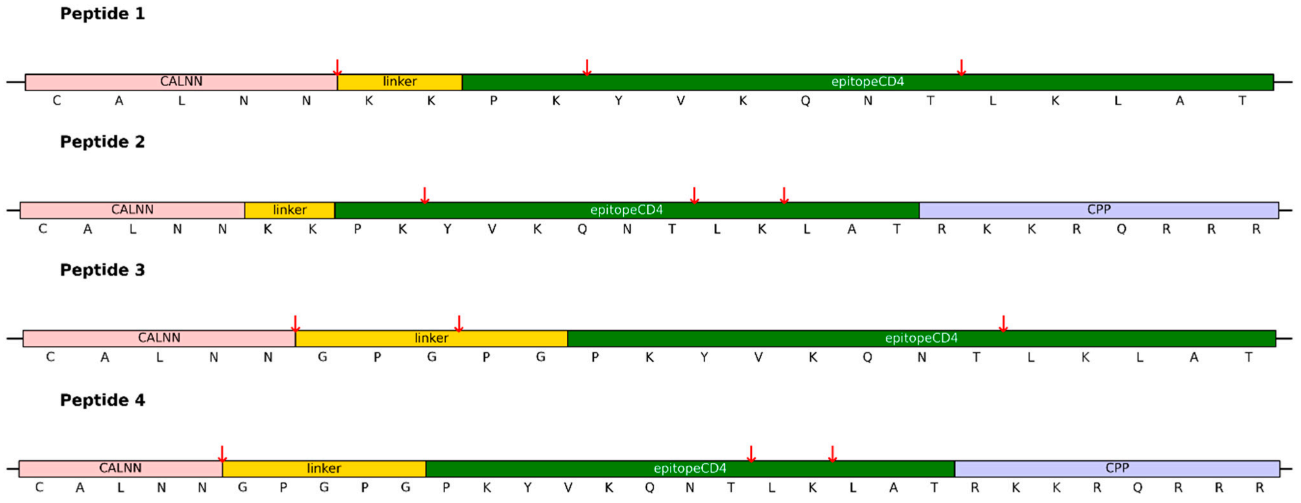

Figure S4. Predictions of cathepsin B cleavage sites in the peptide sequences. The sequences contain the CALNN sequence (pink), linker/spacer (gold), CD4+ epitope from Influenza A hemagglutinin (green) and CPP region (purple). Putative locations of cathepsin B cleavage sites (red arrows) were calculated using cathepsin B specificity matrix from the MEROPS database. Only the top three sites with highest cumulative scored were selected.

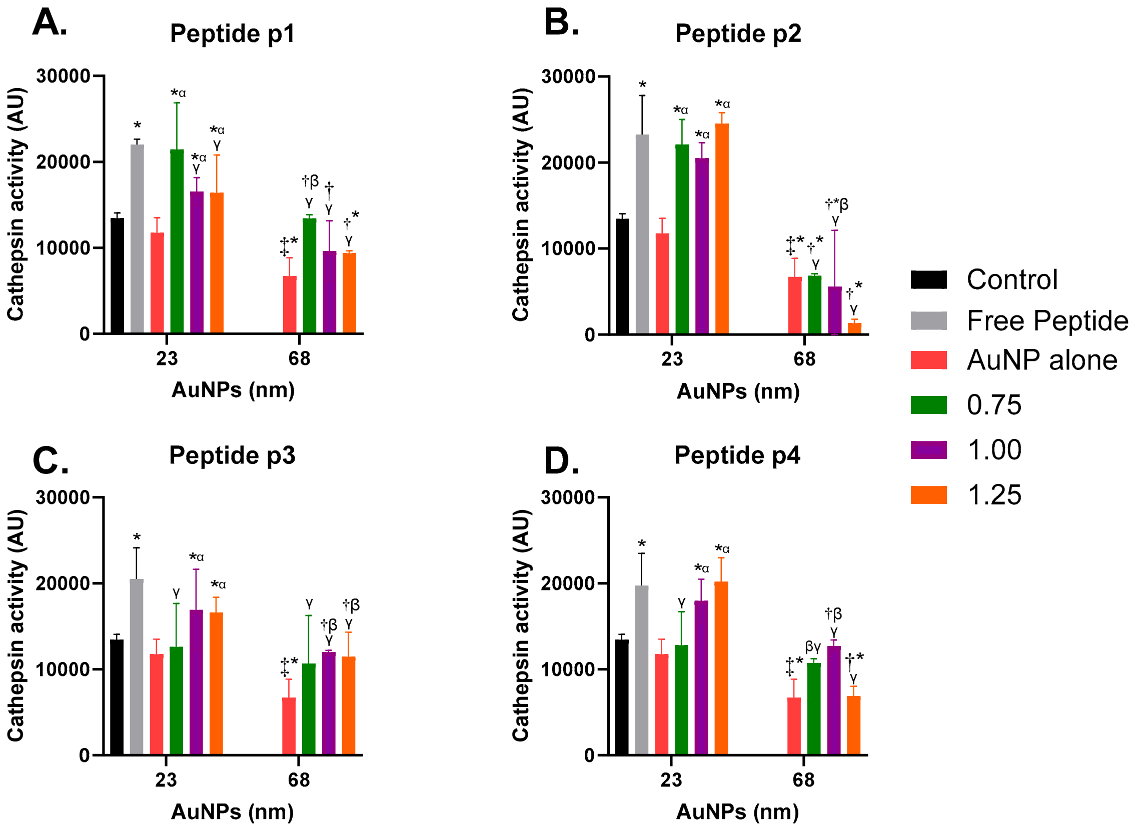

Figure S5. Cathepsin B activity (raw data) measured in macrophages THP-1 incubated with the nanoconjugates. The assays include AuNPs conjugated with peptides (A) p1, (B) p2, (C) p3 and (D) p4), at different surface coverages (0.75, 1.00, and 1.25). Controls correspond to free peptides and free AuNPs. Bars represent the mean ± s.d. (n = 3). *P<0.05 denotes statistically significant differences with respect to control (black column; basal activity of THP-1 macrophages without AuNPs or peptide). αP<0.05 compares with Au23 alone. †P<0.05 between the treatments with p-Au23 and p-Au68 conjugated with the same peptide (comparing same coverages). ‡P<0.05 between treatments with Au23 nanoconjugate and free Au23. βP<0.05 compared with free Au68.
